# A Constitutive Model of High-Early-Strength Cement with Perlite Powder as a Thermal-Insulating Material Confined by Caron Fiber Reinforced Plastics at Elevated Temperatures

**DOI:** 10.3390/polym12102369

**Published:** 2020-10-15

**Authors:** Yeou-Fong Li, Wai-Keong Sio, Tzu-Hsien Yang, Ying-Kuan Tsai

**Affiliations:** 1 Department of Civil Engineering, National Taipei University of Technology, 1, Section 3, Chung-Hsiao E. Rd., Taipei 10608, Taiwan; qoo147896325@gmail.com; 2 Department of Materials and Mineral Resources Engineering, National Taipei University of Technology, 1, Sec. 3, Chung-Hsiao E. Rd., Taipei 10608, Taiwan; yangdavid180@googlemail.com; 3 Department of Environmental Information and Engineering, Chung Cheng Institute of Technology, National Defense University, 75, Shiyuan Rd., Daxi Dist., Taoyuan 335, Taiwan

**Keywords:** high-early-strength cement, perlite, carbon fiber-reinforced polymer, constitutive model, elevated temperature

## Abstract

A parabolic stress–strain constitutive model for inorganic thermal-insulating material confined by carbon fiber-reinforced polymer (CFRP) exposed to a surrounding elevated temperature was proposed in this paper. The thermal-insulating material used in this study was composed of high-early-strength cement (HESC) and perlite powder. The compression strengths of four kinds of perlite powder composition ratios of thermal-insulating materials cylindrical specimens which were confined by one, two, and three-layer CFRP composite materials were acquired. The experimental results showed that the compression strength was enhanced as the amount of perlite substitute decreased or as the number of CFRP wrapping layers increased. The Mohr–Columb failure criteria were adopted to predict the maximum compressive strength of CFRP-confined inorganic thermal-insulating material. The strain at the maximum compressive strength was found from the experimental results, and the corresponding axial strain at the maximum compressive strength in the constitutive model was determined from the regression analysis. Furthermore, the compressive strengths of the four different perlite composites of thermal-insulating materials were obtained when heating the specimens from ambient temperature to 300 °C. The compressive strength decreased with an increase in temperature, and a thermal softening parameter model was proposed; the thermal softening parameter was determined from the experimental maximum compressive strength at an elevated temperature. Combining the above two models, the constitutive model of HESC with perlite powder additive as a thermal-insulating material confined by CFRP under elevated temperature was proposed.

## 1. Introduction

**Figure 1:**
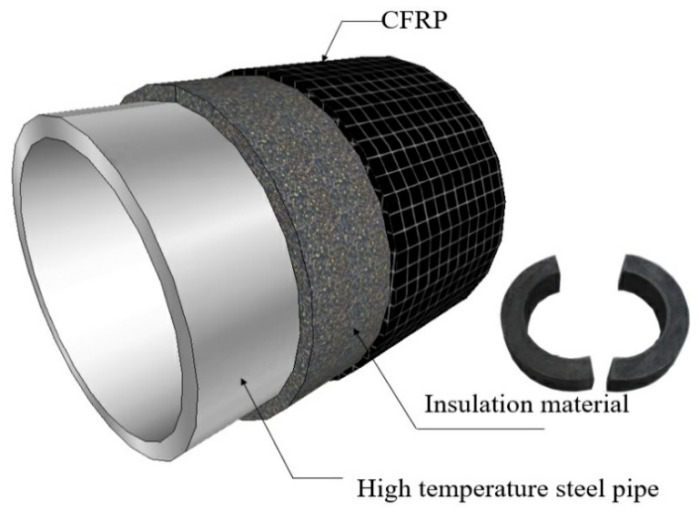
The strengthening method for the high-temperature steel pipelines.

Petrochemicals are critical to the development of the global industry, and low-carbon steel-made pipelines are commonly used for transporting petroleum products because they are the most cost-effective. However, these steel pipes can be corroded, especially in the presence of corrosive acids, sulfur, and chlorides. The common method of steel pipeline repair is to use carbon fiber-reinforced materials or mineral wool-reinforced materials to cover the damaged steel. However, particularly when the damaged or corroded pipelines are assembled under a high-temperature environment, the current strengthening the insulation layer wrapped in the surface of the pipeline is a difficult problem that needs to be overcome. Hence, Li et al. proposed a method to strengthen high-temperature steel pipelines, where the inorganic thermal-insulating materials confined by CFRP are used to strengthen the damaged pipelines settled in high-temperature installations, as shown in Figure 1 [1].

The advantages of the manufacturing process of the CFRP composite material are as follows: high strength, high modulus, corrosion resistance, lightweight, and easy implementation [2,3]. The epoxies are polymer chemical compounds and serve as a bonding element in the CFRP composition. When epoxy resin was exposed to high temperature environment, its mechanical properties will reduce. Therefore, a thermal-insulating material made of HESC with perlite powder additive and wrapped in CFRP on the damaged metallic pipelines was proposed, which is a solution to prolong the lifetime of damaged metallic pipelines under high temperatures. A compressive strength test was performed on specimens made of HESC with various proportions of perlite at different temperatures. This study aims to build a constitutive model of CFRP-confined thermal-insulating material under elevated temperatures.

Perlite powder is an essential material for the application of thermal insulation material. By altering the proportion of perlite additive, it was found that the compressive strength diminished with the increment in the perlite [4,5,6]. The concrete specimen was added with perlite powder, the results indicated that the compressive strength of the concrete specimen diminished while the replacement rate of perlite powder increased. In aspects of the thermal conductivity and mechanical properties of lightweight concrete, the compressive strength and elastic modulus decreased with the increment of lightweight aggregates [7,8,9,10]. Confined concrete constitutive models using FRP and steel jacketing have been researched extensively in the last few decades. The compressive strength models are proposed to calculate the maximum strength of concrete reinforced with steel plate, and fiber-reinforced plastic (FRP) composites. [11,12,13,14,15,16,17,18,19,20,21].

When concrete cylinders and reinforced concrete columns were confined by FRP composite materials, the compressive strength could be enhanced by confining with FRP composite materials [22,23,24,25,26,27]. Instead of cylindrical reinforced concrete columns, the compression strength of square columns can be improved by enclosing with CFRP composite materials as well. The compressive strength increased with the number of CFRP wraps, and the strains of CFRP-confined concrete specimens decreased with the higher inherent strength of the concrete [28]. Rousakis et al. used transversely placed basalt composite rope as an external confining reinforcement and combined this with FRP sheets for reinforced concrete columns with a square section. The results showed that the basalt composite rope could increase the axial strain sustained by the concrete from 2.21% to 5.1% without rope fracture [29,30]. 

As the temperature increased, the behavior of the FRP-confined and unconfined specimens would lead to the failure of the concrete confinement. However, as the compressive strength of the FRP-reinforced concrete is increased, these properties would increase the performance and the effect of the restraining factors of FRP-reinforced concrete [31,32]. Al-Salloum et al. [33] investigated the effect of high temperatures on the performance of concrete externally confined with FRP sheets. The test results showed that the bond strength of the CFRP-overlaid specimens has a significant degradation at a temperature of 200 °C. da Costa Mattos et al. [34] analyzed the glass fiber-reinforced polyurethane repair system for corroded metallic pipelines; it was shown that the water temperature (between 60 and 90 °C) was a major shortcoming in the use of polymeric material in the repair system. 

## 2. Materials and Test Program

In the repair system shown in Figure 1, the materials consisted of HESC, expanded perlite, a carbon fiber sheet, and epoxy resin. The experimental procedures comprised compressive strength tests of thermal-insulating materials made of HESC with various ratios of perlite powder which were confined by CFRP composite materials. Therefore, the maximum compressive strength and the corresponding strain of various numbers of CFRP layers in a specific high-temperature surrounding was proposed.

### 2.1. Materials

HESC was manufactured by the Denka Company in Japan. The components of the HESC were analyzed by utilizing X-ray fluorescence analysis, as shown in Table 1. 

Perlite powder is a white-in-color glassy volcanic rhyolitic lava, which can be expanded to up to 20 times the original dimensions and form a porous morphology when heated above 870 °C. It is commonly called expanded perlite and is a good insulating material due to its porosity characteristics, and it is commonly used in the fields of construction and agriculture and in the chemical industry. The perlite powder was placed on the top sieve, and the sieves were agitated in a mechanical shaker. The sieve analysis test results are shown in Table 2, and the fineness modulus is 2.97.

**Figure 2:**
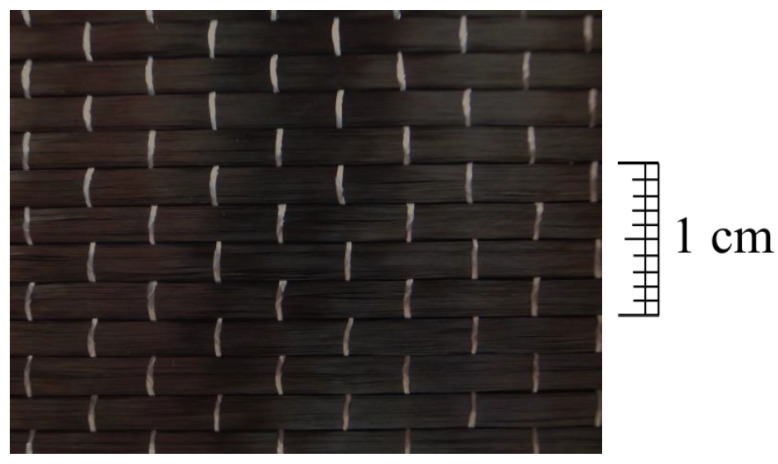
Carbon fiber sheet.

CFRP composite material is made of uni-directional carbon fiber sheet and polymer, where the carbon fiber provides a higher tensile strength and stiffness and the polymer adhesive material offers a cohesive strength to hold the fibers together. The uni-directional carbon sheet uses 12,000 carbon fiber monofilaments in a carbon fiber bundle, and its fiber area weight (FAW) is 300 g/m^2^. The material properties and specification of the uni-directional carbon fiber sheet and epoxy resin are listed in Table 3 and Table 4, respectively. A photo of the uni-directional CFRP sheet is shown in Figure 2. 

### 2.2. Testing Program

This study conducted compressive testing of cylindrical and cubic specimens, utilizing a 981 kN universal testing machine at the Department of Civil Engineering, National Taipei University of Technology. The compressive testing required the specimens to be placed in the universal testing machine, and a load cell (WF 17120, Wykeham Farrance, Milan, Italy) with a 490 kN capacity and strain gauges (KFGS-20-120-C1 L3M2R, KYOWA, Tokyo, Japan) were utilized. According to American Society for Testing and Materials (ASTM) C39/C39M-18 [35], the loading rate of each specimen was 900~1800 N/s. Additionally, a data acquisition system (KL-10, Geomaster Group, Tianjin, China) was used to obtain the force-displacement information during compressive testing.

Besides this, perlite powder of proportions 10, 20, and 30 wt.% was added to the HESC, and the water–cement ratio of HESC was 0.35. According to ASTM C109/C M109-02 [36], the compressive strength test was conducted on a standard cubic specimen with the dimensional aspects of 5 × 5 × 5 cm. The cubic specimen, after 28 days of curing for the compressive strength testing, was examined under various temperatures of 100, 150, 200, 250, and 300 °C. For the compressive strength test, the specimens were placed to heat up on the crucible in the furnace, then withdrawn out from the furnace immediately while we waited for the specimens to reach the above-mentioned temperature, then we started to proceed with the compressive testing.

Table 5 shows the specification of the specimen name and test method, where SPC represents the insulation material specimens with perlite mixed for compression testing, SPCC represents insulation material specimens with the perlite additive confined by CFRP composites for compression testing, and SPTC represents the insulation material specimens for compression testing under elevated temperature conditions. Table 6 shows the lists of the mixed ratios of the thermal-insulating materials.

Table 7 lists the identification and number of cylindrical specimens. A total number of 48 cylindrical insulation material specimens were tested to obtain the compression strength of the specimens confined by different numbers of CFRP layers. 

The application procedures of the CFRP composite materials to the cylindrical specimens were as follows: Before applying the CFRP layer with a coating epoxy adhesive, we cleaned the surface of the cylinders to ensure a good adhesion at the interfaces. Firstly, we applied a thin layer of primer epoxy resin to the surface of the cylinder. After the primer epoxy resin was cured at room temperature for a few hours, a carbon fiber sheet was applied to paste onto the cylindrical specimens. The epoxy was applied by using a paintbrush layer by layer for a fully saturated infiltration on the carbon fiber. The overlapping cover length was more than 10 cm, and the next layer was applied 24 h later. After applying the desired sheet, the CFRP jacketing was cured at room temperature.

**Figure 3:**
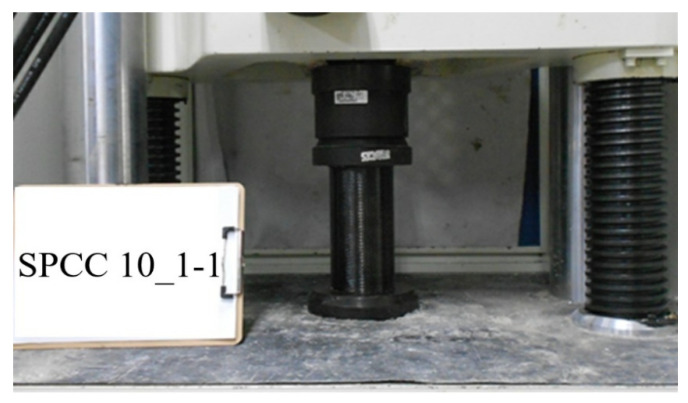
Compressive strength test for the cylindrical CFRP-confined specimen.

The tensile circumferential strain of CFRP was measured by strain gauges (KFGS-20-120-C1 L3M2R, Kyowa, Japan) attached to the middle surface of the CFRP-confined specimen. The concrete cylinder confined by the CFRP composites established on the compression testing instrument is shown in Figure 3.

Similarly, a total number of 72 cubical specimens (5 × 5 × 5 cm) were made to investigate the compression strength of thermal-insulating materials at different elevated temperatures, as shown in Table 8.

## 3. Constitutive Model of the CFRP-Confined Thermal-Insulating Material

The following subsections will introduce the results of the compressive strength test of the thermal-insulating materials without and with CFRP confinement. The research aims at studying the effect of various amounts of perlite additive (0, 10, 20, and 30 wt.%) on the compressive strength.

### 3.1. Compressive Strength Test of SPCs

A series of compressive strength tests were carried out on SPC specimens to observe the changes in terms of strength related to the different structural designs with various components of the composites. Table 9 shows that the compressive strength diminishes as the addition of perlite content rises.

### 3.2. Compression Test on CFRP-Confined Specimens

In this subsection, the compressive strength and failure mode of the SPC and SPCC specimens confined by different layers of CFRP composite material were investigated.

#### 3.2.1. HESC Specimens

**Figure 4:**
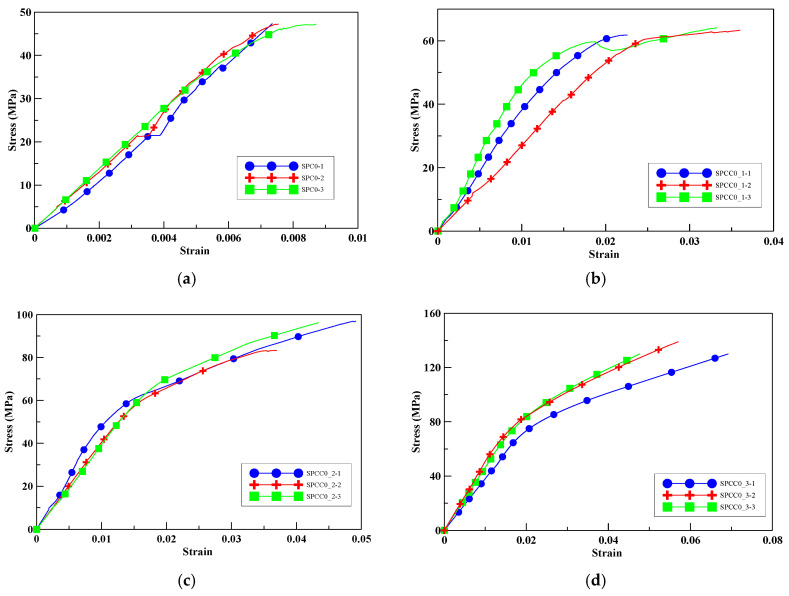
Stress–strain curves of specimens (**a**) SPC0, (**b**) SPCC0_1, (**c**) SPCC0_2, and (**d**) SPCC0_3.

The compression test was conducted with a cylindrical specimen without perlite as a control group. Figure 4a shows the relationship between the axial stress–axial strain relationships of the cylindrical specimens without CFRP confinement. The average maximum compressive strength of SPC0 was 42.75 MPa. The maximum compressive strength of specimens SPCC0_1, SPCC0_2, and SPCC0_3 increased by 33%, 95%, and 181%, respectively. The maximum compressive strength and corresponding strain of the specimens increased with increasing the number of CFRP wrapping layers, as shown in Figure 4b–d. In SPCC0_2. It should be noted that the strength was suddenly drop due to the partial fracture occurrence of CFRP while the loading stress was about 60 MPa.

#### 3.2.2. HESC with Perlite Content

From the compressive test results, the average maximum compressive strength without wrapping CFRP on a cylinder consisting of 10, 20, and 30 wt.% perlite additive was 26.04, 17.18, and 12.84 MPa, respectively. As revealed in Table 10, the number of CFRP layers increased for the confined specimens; the maximum compressive strength of the confined specimens with 10, 20, and 30 wt.% of added perlite increased by 51.1~219%, 78.4~340%, and 107.6~439.1%, respectively. As the number of CFRP layers increased, especially in specimen SPCC30_3, the compressive strength increased the percentage of intensity up to 439.1%.

According to the test data, the maximum compressive strength of the SPCC specimens was improved due to the supported confinement of a different number of CFRP layers. As the number of CFRP layers increased for the confined specimens, no matter what the percentage of perlite additives was, the increasing percentage of maximum compressive strength could be improved. Table 11 shows the post-test photographs of SPCC specimens; it can be seen that the fracture in CFRP and the spalling in concrete are more severe as the number of confining layers increases. The measured lateral strain at the maximum compressive strength of SPCCs is listed in Table 12.

### 3.3. Constitutive Model for CFRP-Confined Thermal-Insulating Material

This study used both the analytical model and regression analysis of experimental data to progress a constitutive model that was appropriate for representing the compression behavior of thermal-insulating material specimens with a different number of CFRP layers. The maximum compressive strength of the thermal-insulating materials confined by CFRP was derived from the Mohr–Coulomb failure criterion; the corresponding strain at the maximum compressive strength was derived from the regression analysis of the data.

#### 3.3.1. Compressive Stress–Strain Relationship of the Constitutive Model

**Figure 5:**
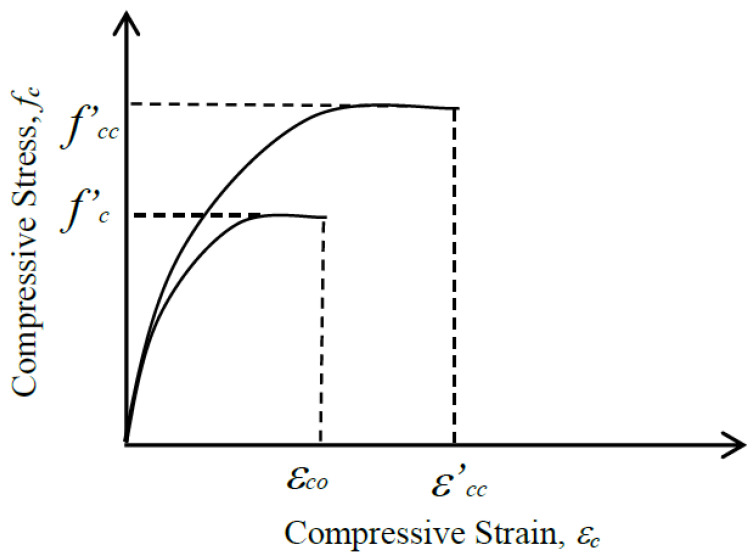
The stress–strain relationships of the concrete cylinders with and without CFRP.

The proposed parabolic constitutive model for the CFRP-confined specimens is shown in Figure 5. In rock mechanics, the Mohr–Coulomb failure criterion is often used and is expressed as the following equation:

(1) $$\sigma_{1}=C_{0}+\sigma_{3}\times \tan^{2}(45^{{}^{\circ}}+\frac{\phi }{2}),$$

where $\sigma_{1}$ is the uniaxial compressive strength of rock with $\sigma_{3}$ lateral confinement stress, ϕ is the internal friction angle, and $C_{0}=S_{0\;}\times \tan(45^{{}^{\circ}}+\phi /2)$ is the uniaxial compressive strength without lateral confinement [37].

The mechanical behavior of thermal-insulating material confined by the CFRP composite material is similar to that of rock confined by lateral water pressure. Therefore, the maximum compressive strength of the proposed model was adopted from the Mohr–Coulomb failure criterion, and can be expressed as follows:

(2) $$f_{cc}^{’}=f_{c}^{’}+f_{l}^{’}\times \tan^{2}(45^{{}^{\circ}}+\frac{\phi }{2}),$$

(3) $$f_{l}^{’}=\frac{2\times n\times t\times E_{cf}\times \varepsilon_{cf}\times k_{c}}{D}.$$

Where:

*f’_cc_* = the compressive strength of the confined concretes;

*f’_c_* = the compressive strength of the unconfined concretes;

*f’_l_* = the effective lateral confined stress of CFRP;

*ϕ* = internal friction angle of concretes;

*n* = the number of CFRP wrapping layers;

*t* = the thickness of a single CFRP layer;

*E_cf_* = the elastic modulus of CFRP;

*ε_cf_* = the strain of CFRP;

*k_c_* = a sectional shape factor.

In this paper, the internal friction angle was proposed as follows:

(4) $$\phi =A^{{}^{\circ}}+B^{{}^{\circ}}(\frac{f_{c}^{’}}{f_{l}^{’}})\;\leq 45{}^{\circ}.$$

By applying Equation (3), the lateral confined stress of the CFRP confinement can be derived. The measured lateral strains of the cylindrical specimens wrapped with different numbers of CFRP sheets are listed in Table 12, and the average measured lateral strain was *ε_cf_* = 1.1%. For one layer, two layers, and three layers of CFRP-confined cylindrical specimens (Φ10 cm × 20 cm), the effective lateral confined stresses were 7.84, 15.68, and 23.51 MPa, respectively.

To find the angle of internal friction, Equation (3) can be modified as follows:

(5) $$\frac{f_{cc}^{’}}{f_{c}^{’}}=1+\frac{f_{l}^{’}}{f_{c}^{’}}\times \tan^{2}(45{}^{\circ}+\frac{\phi }{2}).$$

**Figure 6:**
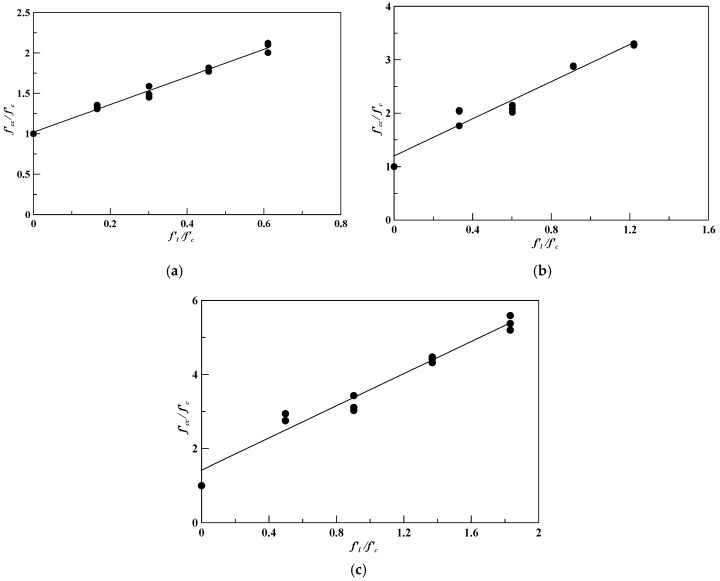
The regression analysis results of the specimens confined with different layers of CFRP with the friction angle: (**a**) one-layer CFRP (*ϕ* = 15.2°); (**b**) two-layer CFRP (*ϕ* = 15.6°); (**c**) three-layer CFRP (*ϕ* = 21.7°).

In Equation (5), a regression analysis was performed to obtain the experimental internal friction angle, *ϕ*, where *f’_l_/f’_c_* and *f’_cc_/f’_c_* are set as the abscissa and ordinate axes of the coordinates. The computed results are shown in Figure 6. The experimental values of the internal friction angles were 15.2°, 15.6°, and 21.7°, for the specimens confined with different layers of CFRP, as shown in Figure 6a–c, respectively.

A regression analysis was performed to derive the coefficients A and B in Equation (4), where *f’_c_/f’_l_* and *ϕ* were set as the abscissa and ordinate axes of coordinates; the values of *f’_c_/f’_l_* used for the analysis are listed in Table 13.

The results of the coefficients A and B were 19.4 and −0.95; thus, Equation (4) was rewritten as:

(6) $$\phi =19.4^{{}^{\circ}}-0.95^{{}^{\circ}}(\frac{f_{c}^{’}}{f_{l}^{’}}).$$

**Figure 7:**
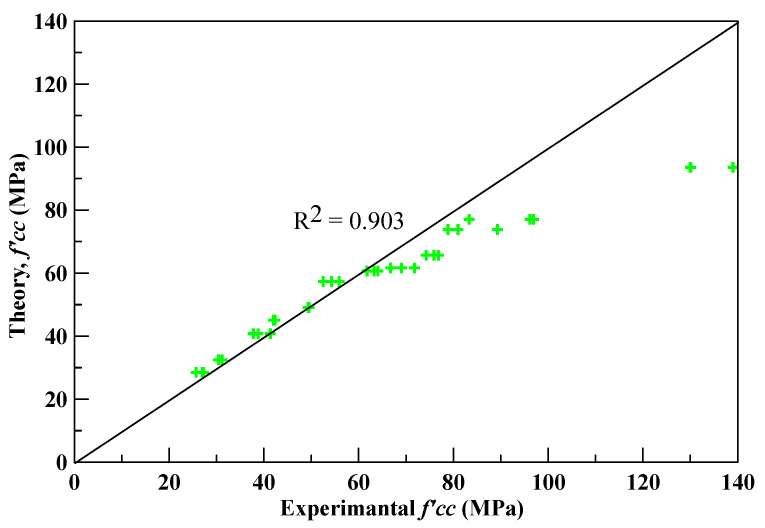
The deviation and correlation coefficient of the analytical and experimental maximum compressive strengths.

By substituting the above-mentioned internal friction angles (*ϕ*) into Equation (2), the analytical maximum compressive strengths could be obtained. The maximum compressive strengths of the experimental and analytical results are shown in Table 14, where the average absolute error is 7.24%. Figure 7 shows the deviation between the experimental and analytical maximum compressive strengths, as well as the correlation coefficient (R^2^) is 0.903. The results show that the proposed constitutive model could portend the experimental maximum compressive strength with a realistic degree of accuracy.

#### 3.3.2. The Axial Strain at the Maximum Compressive Strength

As the axial stress achieves the maximum compressive strength “$f_{cc}^{’}$”, the CFRP breaks and fails to provide confinement, and the corresponding axial strain at the maximum compressive strength “$\varepsilon_{cc}^{’}$” is primarily governed by the CFRP confining strength. Therefore, the analytical $\varepsilon_{cc}^{’}$ could be presented as the following equation.

(7) $$\varepsilon_{cc}^{’}=\varepsilon_{co}\left[1+\alpha \tan^{2}(45^{{}^{\circ}}+\frac{\phi }{2})\frac{f_{l}^{’}}{f_{c}^{’}}\right].$$

**Figure 8:**
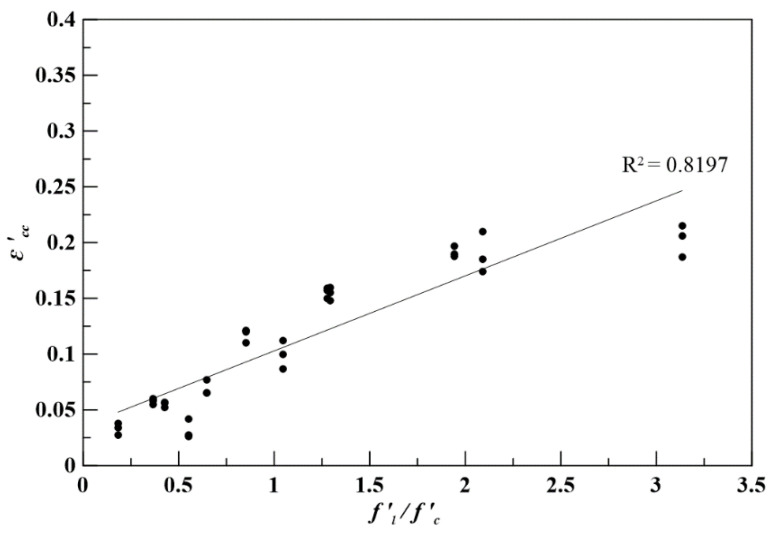
The $\varepsilon_{cc}^{’}$ and($f_{l}^{’}/f_{c}$) relationships of the constitutive model and experimental consequences.

From the regression analysis, the parameters $\varepsilon_{co}$ and α can be found, where $\varepsilon_{co}$ = 0.0356 and α = 0.056. In Figure 8, the experimental data are expressed as dots which are obtained from the stress–strain relationships of specimen SPCCs, and the straight line is obtained from Equation (7). It shows that Equation (7) can anticipate the corresponding axial strain at the maximum strength by anticipating consistency as well.

Then, the compressive stress–strain relationship of the constitutive model, as shown in Figure 5, can be expressed as follows.

(8) $$f_{c}=f_{cc}^{’}\left[-{\left(\frac{\varepsilon_{c}}{\varepsilon_{cc}^{’}}\right)}^{2}+2\left(\frac{\varepsilon_{c}}{\varepsilon_{cc}^{’}}\right)\right],\;\mathrm{where}\;0\leq \varepsilon_{c}\leq \varepsilon_{cc}^{’}.$$

In Equation (8), *f_c_* and *ε_c_* are the compression stress and strain of the specimens wrapped with CFRP composite material.

## 4. Constitutive Model of the Thermal-Insulating Material at Elevated Temperature

The purpose of the experiment was to understand the compressive strengths of the thermal-insulating material specimens (with the addition of 0, 10, 20, and 30 wt.% perlite powder) at different temperature conditions of 25, 100, 150, 200, 250, and 300 °C. These were applied to standard cubic specimens with a size of 5 × 5 × 5. The specimens were settled on a crucible to heat in the oven. While the specimens were taken out of the furnace, the compressive strength test was performed immediately when the scheduled temperature was achieved. The thermal softening model was proposed for the maximum compressive strength. According to the experimental results, the thermal softening parameters of the maximum compressive strength were determined.

### 4.1. Compression Test at Elevated Temperature Conditions

**Figure 9:**
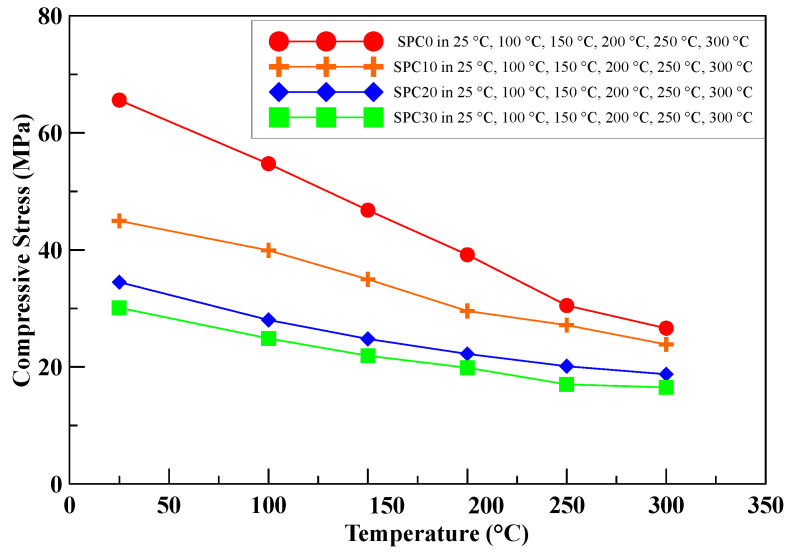
The maximum compressive strength of the specimens with various perlite ratios at different elevated temperatures.

The relationship of the compressive strength of the specimens with various perlite ratios and different temperatures is shown in Figure 9. The results show that the maximum compressive strength of the thermal-insulating material specimens also diminishes as the amount of perlite added rises. The maximum compressive strength was reduced by increasing the content of the perlite additive with the rising temperature.

The results showed that the weight ratio of perlite added played an important role as a thermal-insulating material for resisting the thermal transport during the elevated temperatures. For the compressive strength test at a specific temperature, although the specimen consisted of a lower compression strength with a higher proportion of perlite added, the difference in the compression strength of the specimens with perlite added for comparing between 0 wt.% and 30 wt.% decreased with the elevating temperature.

### 4.2. A Maximum Compressive Strength Model at Elevated Temperatures

For the maximum compressive strength of the thermal-insulating material at elevated temperatures, an exponential thermal softening model was proposed as follows [38,39]:

(9) $$f_{T}^{’}=f_{c}^{’}\times e^{-\lambda (T-T_{ref})},$$

where *f’_T_* and *f’_c_* are the maximum compressive strengths of the thermal-insulating material under an elevated temperature (*T*) and the reference temperature (*T_ref_*), respectively. In Equation (9), *λ* is the thermal softening parameter of the thermal-insulating materials with various perlite ratios added.

**Figure 10:**
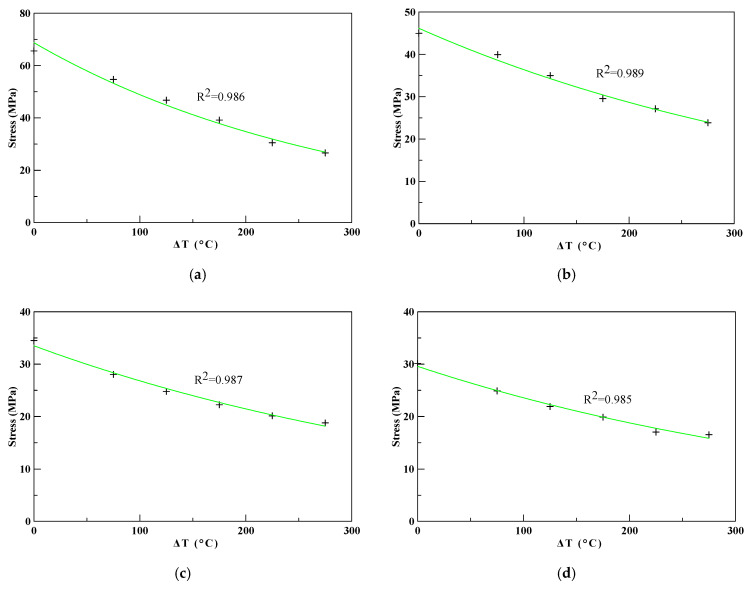
The regression analysis results of the thermal softening parameter of HESC with (**a**) 0% perlite (*λ =* 0.00341), (**b**) 10% perlite (*λ =* 0.00238), (**c**) 20% perlite (*λ =* 0.00223), (**d**) 30% perlite (*λ =* 0.00229).

The results of the compressive strength test with various perlite ratios at different elevated temperatures were analyzed numerically via regression analysis to obtain a thermal softening parameter. Figure 10 shows the results of the regression analysis results of the thermal softening parameter of HESC with different ratios of perlite powder. The thermal softening parameters of the thermal-insulating materials obtained from the regression analysis were *λ =* 0.00341, 0.00238, 0.00223, and 0.00229 for perlite ratios of 0, 10, 20, and 30 wt.%, respectively.

The thermal softening material parameter of the thermal-insulating material with different perlite ratios in weight was substituted into Equation (9) to obtain the analytical maximum compressive strength. The absolute errors between the analytical and experimental maximum compressive strengths are listed in Table 15, and the average absolute errors were 5.12%, 3.13%, 3.49%, and 2.63% for perlite ratios of 0, 10, 20, and 30 wt.%, respectively. The correlation coefficients (*R^2^*) between the analytical and experimental maximum compressive strengths were found to be 0.95, 0.98, 0.98, and 0.98 for perlite ratios of 0, 10, 20, and 30 wt.%, respectively. The proposed thermal softening model, as shown in Equation (9), could predict the experimental maximum compressive strength with a reasonable degree of accuracy.

## 5. Constitutive Model of CFRP-Confined Thermal-Insulating Material at Elevated Temperatures

This study used both the theoretical Mohr–Coulomb failure criterion and a regression analysis of the experimental data to develop a constitutive model of CFRP-confined thermal-insulating material at elevated temperatures. The maximum compressive strength of the CFRP-confined thermal-insulating materials was gained from the Mohr–Coulomb failure criterion. In the meantime, the corresponding axial strain at the maximum compressive strength was derived from the test results via regression analysis. Therefore, a parabolic stress–strain relationship constitutive model for CFRP-confined specimens was proposed, as shown in Equation (8). For the maximum compressive strength of the thermal-insulating material at elevated temperatures, a thermal softening model was proposed, as shown in Equation (9).

Eventually, the physical-based constitutive model (stress–strain relationship) for thermal-insulating material specimens wrapped with CFRP under an elevated temperature is proposed as follows.

(10) $$f_{T}=f_{cc}^{’}\times e^{-\lambda (T-T_{ref})}\left[-{\left(\frac{\varepsilon_{c}}{\varepsilon_{cc}^{’}}\right)}^{2}+2\left(\frac{\varepsilon_{c}}{\varepsilon_{cc}^{’}}\right)\right],\;\mathrm{where}\;0\leq \varepsilon_{c}\leq \varepsilon_{cc}^{’},$$

where *f_T_* and *ε_c_* are the compression stress and strain of specimens wrapped with CFRP under elevated temperatures. Then, a parabolic stress–strain relationship of the constitutive model for CFRP-confined thermal-insulating material at elevated temperatures was proposed, as shown in Equation (10).

## 6. Conclusions

This study presented a constitutive model of CFRP-confined thermal-insulating material at elevated temperatures. From the experimental and regression analysis results, the following conclusions were drawn:

1. Based on the compressive test results, the maximum compressive strength of HESC diminished with the increment in the addition of perlite, and also decreased with elevating temperature.
2. From the compressive test results, the maximum compressive strength of the thermal-insulating material confined by CFRP was enhanced with an increment in the number of CFRP layers used.
3. For the maximum compressive strength prediction model, the average absolute error between the analytical model and the experimental maximum compressive strengths was 7.24%, and its correlation coefficient (*R^2^*) is 0.903.
4. For the proposed thermal softening model of the thermal-insulation specimens at elevated temperature, the average absolute errors between the analytical and experimental maximum compressive strengths errors were 5.12%, 3.13%, 3.49%, and 2.63% for perlite ratios of 0, 10, 20, and 30 wt.%, respectively. The correlation coefficients (*R^2^*) between the analytical and experimental maximum compressive strengths errors were 0.95, 0.98, 0.98, and 0.98 for perlite ratios of 0, 10, 20, and 30 wt.%, respectively.
5. The physics-based constitutive model for inorganic thermal-insulating material confined by CFRP exposed at a surrounding elevated temperature was proposed, and this proposed constitutive model can predict the experimental maximum compressive strength for the thermal-insulating material confined by CFRP composite materials accompanied with increasing temperature.

## Figures and Tables

**Table 1:** Components of the HESC material.

Composition	Percentages (%)
CaO	68.4
SiO_2_	11.9
Al_2_O_3_	9.20
SO_2_	5.10
Fe_2_O_3_	2.90
K_2_O	1.80
TiO_2_	0.70
P_2_O_5_	0.50

**Table 2:** Sieve analysis test results of the perlite powder.

Sieve Number	Weight Retained (g)	Percent Retained	Percent Coarser Than
#4	0	0	0
#8	0	0	0
#16	106	0.53	53.27
#30	31	0.16	68.84
#50	26	0.13	81.91
#100	22	0.11	92.96
Pan	2	0.01	100.00
			Fineness modulus (F.M.) = 2.97

**Table 3:** Material properties of the uni-directional carbon fiber sheet (12 k, FAW = 300 g/m^2^).

**Uni-directional Carbon Fiber Sheet**	**Specification**	
	Young’s modulus	250 (GPa)
	Tensile strength	4.9 (GPa)
	Thickness	0.16 (mm/layer)
	Ultimate strain	0.02

**Table 4:** Material properties of the epoxy resin.

**Epoxy Resin**	**Specification**	
	Viscosity (at 25 °C)	1823 (cps)
	Young’s modulus	3.5 (GPa)
	Tensile strength	52.2 (MPa)
	Tensile adhesive strength	10.5 (MPa)

**Table 5:** The unconfined specimens’ dimensions and their corresponding test method.

Specimen	Description (Test Method)	Dimension	Perlite Ratio in Weight (%)
SPC	HESC with perlite (ASTM C39/C39M-18)	Φ10 cm × 20 cm	0; 10; 20; 30
SPCC	HESC with perlite confined by CFRP (ASTM C39/C39M-18)	Φ10 cm × 20 cm	
SPTC	HESC with perlite under elevated temperature (ASTM C109/C M109-02)	5 cm × 5 cm × 5 cm	

**Table 6:** Designed different perlite ratios of the thermal-insulating material specimens.

Specimen	Perlite Ratio in Weight (%)	Perlite (g)	HESC (g)	Water (g)
SPC0	0	0	2000	700
SPC10	10	200	1800	630
SPC20	20	400	1600	560
SPC30	30	600	1400	490

**Table 7:** The number of test cylinders with a different number of CFRP layers.

Specimen	Shape	No. of CFRP Layer	No. of Cylindrical Specimen
SPC0	Cylinder	Nil, 1, 2, 3	12
SPCC10			12
SPCC20			12
SPCC30			12

The total number of specimens: 48.

**Table 8:** The number of cubic test specimens at elevated temperatures.

Specimen	Shape	Elevated Temperatures (°C)	No. of CFRP Cubic Specimen
SPTC0	Cube	25, 100, 150, 200, 250, 300	18
SPTC10			18
SPTC20			18
STPC30			18

The total number of specimens: 72.

**Table 9:** Compressive strength test results of the perlite powder additive.

Specimen	Perlite Ratio in Weight (%)	Average Compressive Strength (MPa)	Decrease Percentage (%)
SPC0	0	47.25	-
SPC10	10	26.04	44.89
SPC20	20	17.18	63.64
SPC30	30	12.84	72.82

**Table 10:** Compressive strength test results for the SPCC specimens.

Specimen	No. of CFRP Layers	Average Maximum Compressive Strength (MPa)	Increase Percentage (%)
SPCC0_1	1	63.05	33.4
SPCC0_2	2	92.15	95.0
SPCC0_3	3	133.04	181.6
SPCC10_1	1	39.31	51.1
SPCC10_2	2	54.25	108.3
SPCC10_3	3	83.07	219.0
SPCC20_1	1	30.69	78.4
SPCC20_2	2	49.46	187.6
SPCC20_3	3	75.68	340.0
SPCC30_1	1	26.66	107.6
SPCC30_2	2	42.19	228.6
SPCC30_3	3	69.22	439.1

**Table 11:** Post-test photos of SPCCs.

Specimen with Perlite Percentage (%)	1-Layer CFRP	2-Layer CFRP	3-Layer CFRP
Without perlite (SPCC0)	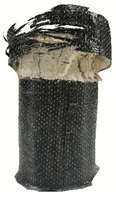	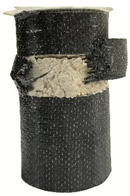	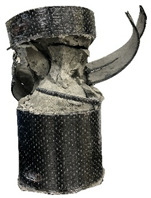
10% perlite (SPCC10)	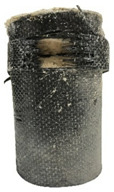	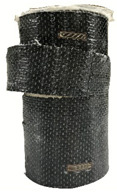	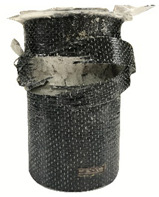
20% perlite (SPCC20)	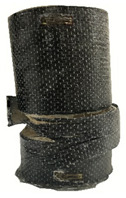	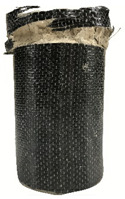	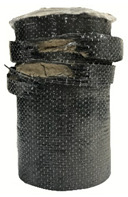
30% perlite (SPCC30)	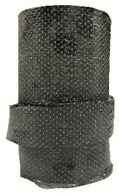	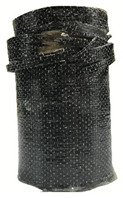	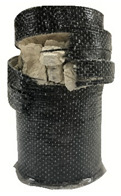

**Table 12:** The measured lateral strains at the maximum compressive strength of SPCCs.

Strain	1-Layer CFRP SPCC0-1	2-Layer CFRP SPCC0-2	3-Layer CFRP SPCC0-3
Measured Lateral Strain (%)	0.898	1.092	1.223
	0.975	1.076	1.173
	1.186	1.125	1.159
Average Lateral Strain (%)	1.020	1.098	1.185

**Table 13:** The *f’_c_/f’_l_* values for a different amount of perlite ratios and the number of CFRP layers.

		Perlite Ratio in Weight			
	No. of CFRP Layers	0%	10%	20%	30%
$\frac{f_{c}^{’}}{f_{l}^{’}}$ Value	1	6.03	3.32	2.19	1.64
	2	3.01	1.66	1.10	0.82
	3	2.01	1.11	0.73	0.55

**Table 14:** The experimental and analytical compressive maximum strengths.

Specimen	Number of CFRP Layers	Average Experimental Maximum Strength (MPa)	Analytical Maximum Strength (MPa)	Absolute Error (%)
SPCC10_1	1	39.31	40.80	3.79
SPCC20_1	1	30.69	32.54	6.03
SPCC30_1	1	26.66	28.50	6.90
SPCC10_2	2	54.25	57.33	5.68
SPCC20_2	2	49.46	49.10	0.73
SPCC30_2	2	42.19	45.08	6.85
SPCC10_3	3	83.07	73.90	11.04
SPCC20_3	3	75.68	65.68	13.21
SPCC30_3	3	69.22	61.66	10.92

Average absolute error (%) 7.24.

**Table 15:** Absolute errors and correlation coefficients between the experimental and analytical results.

-	*ΔT* (°C)	Perlite Ratio in Weight (%)			
-		0	10	20	30
Absolute Error (%)	0	0	0	0	0
	75	9.10	5.84	4.08	2.02
	125	6.51	7.51	1.64	10.01
	175	3.96	4.85	3.78	11.72
	225	8.39	4.57	5.40	3.56
	275	0.99	1.64	5.34	5.59
Average Absolute Error (%)	-	5.12	3.13	3.49	2.63
Correlation Coefficient (*R^2^*)	-	0.95	0.98	0.98	0.98
